# Spatiotemporal evolution and influencing factors of China’s economic development performance under carbon emission constraints

**DOI:** 10.1186/s13021-023-00235-z

**Published:** 2023-08-11

**Authors:** Zhixiang Xie, Rongqin Zhao, Liangang Xiao, Minglei Ding

**Affiliations:** 1 College of Surveying and Geo-Informatics, North China University of Water Resources and Electric Power, Zhengzhou, 450046 China; 2 Key Laboratory of Geospatial Technology for Middle and Lower Yellow River Regions (Henan University), Ministry of Education, Kaifeng, 475004 China

**Keywords:** Carbon emission constraints, Economic development performance, Technical efficiency, Malmquist productivity index, Total factor productivity, Tobit regression model

## Abstract

**Background:**

China’s high-quality economic development depends on achieving sustainable economic development, reaching peak carbon emissions, achieving carbon neutrality, and intensifying the development of an industrial and energy structure that saves resources and protects the environment. This study used the data envelopment analysis (DEA) model and the Malmquist productivity index to measure the economic development performance of mainland China under carbon emission constraints. Then, it described the spatiotemporal evolution of economic development performance and analyzed its influencing factors using the Tobit model.

**Results:**

The results revealed that there were obvious differences in the trends of the static and dynamic performance of economic development. On the one hand, the static performance of economic development exhibited an upward trend from 2008 to 2020. Its distribution characteristics were dominant in the higher and high-level areas. On the other hand, the dynamic performance had a downward trend from 2008 to 2016 and then an upward trend from 2016 to 2020. In most provinces, the dynamic performance was no longer constrained by technological progress but rather by scale efficiency. It was found that the main factors influencing economic development performance were urbanization level, energy efficiency, vegetation coverage, and foreign investment, while other factors had no significant influence.

**Conclusions:**

This study suggests that China should improve its economic development performance by increasing the use of clean energy, promoting human-centered urbanization, increasing carbon absorption capacity, and absorbing more foreign capital in the future.

## Background

Since its economic reform, China has experienced consistent and positive economic development. However, it is difficult to maintain the traditional economic development model characterized by high input, low benefit, low added value, and dependence on export-oriented products for a long period. Problems such as inadequate effective supply, low input–output, tight environmental constraints, and lack of sustainability have hindered the transformation of China’s economy toward a path of high-quality development [1].

The excessive emission of greenhouse gases leads to the greenhouse effect, which has a negative impact on the climate. Carbon dioxide is the main component of greenhouse gases. Therefore, reducing its emissions is a necessary means to solve climate problems [2–4]. To reduce greenhouse gas emissions, many countries signed the Paris Agreement and committed to an ambitious goal of limiting the rise of global average temperature from pre-industrial levels to 2 °C, preferably 1.5 °C during the latter half of this century [5]. In 2020, the Chinese government announced that it would implement stronger policy measures and strive to achieve the peak of carbon emissions before 2030 and carbon neutrality before 2060 [6, 7].

In recent years, a low-carbon economy has become a necessity for China in its efforts to achieve high-quality economic development and reduce greenhouse gas emissions, especially due to improvements in energy conservation, carbon reduction, and green development. Low-carbon economic development is guided by the principles of sustainable development, driven by technological innovation, and facilitated through industrial structure upgrades and new energy sources. Its goal is to reduce fossil fuel consumption and greenhouse gas emissions to achieve a mutually beneficial relationship between economic development and environmental protection [8]. On the one hand, low-carbon economic development reflects the efforts of the Chinese government in tackling climate change. On the other hand, it represents a necessary measure in achieving high-quality economic development in China. Therefore, it is imperative to evaluate the economic development performance of China under carbon emission constraints and identify strategies for its improvement. Researchers have carried out a large number of studies on the topic of a low-carbon economy, concentrating on defining it, measuring its development level, and analyzing its influencing factors and development paths. The concept of a low-carbon economy was introduced in 2003 by the British government in its Energy White Paper, “Our Energy Future: Creating a Low-carbon Economy.” It defined a low-carbon economy as a means of improving people’s living standards and quality of life through higher efficiency in resource utilization, that is, achieving higher output with lower resource consumption and pollution [9–11]. Feng and Niu [12] believe that a low-carbon economy encompasses various economic forms, such as low-carbon development, low-carbon industry, low-carbon technology, and low-carbon lifestyle. Furthermore, a low-carbon economy is characterized by low energy consumption, low pollution, and low emissions. It aims to meet the basic requirements of carbon-based energy in addressing global warming and achieving sustainable economic and social development. To evaluate the low-carbon economy, researchers have developed comprehensive evaluation index systems based on the concept and connotation of a low-carbon economy. To measure the development level of the low-carbon economy on the national, provincial, and urban levels, researchers have employed quantitative analysis methods, such as the analytic hierarchy process (AHP) [13, 14], entropy weight method [15], factor analysis method [16], driving-pressure-state-impact-response (DPSIR) model [17, 18], technique for order preference by similarity to an ideal solution (TOPSIS) model [19], data envelopment analysis model [20–22], low-carbon economic dispatch (LCED) model [23], and super-slack based measure (super-SBM) model [24]. Shimada et al. [25] simulated the level of low-carbon economic development of Shiga Prefecture in Japan under different future scenarios. They found that the land use pattern, promotion of new energy sources, and lifestyle are the main influencing factors in the development of a low-carbon economy. Liu and Zhao [26] used the interpretive structural model (ISM) to obtain the influencing factors of low-carbon economic development in China. It was found that extensive economic development, a lack of awareness among residents, and a shortage of professionals were the key obstacles to low-carbon economic development. To choose an appropriate development path for a low-carbon economy, the existing studies are mainly conducted from two perspectives. First, they analyzed the concept of a low-carbon economy to propose measures for carbon reduction and those for efficiency enhancement of economic development [27–29]. Second, they examined empirical cases to identify the key factors hindering the development of the low-carbon economy and provide personalized regulation strategies [30, 31].

Several limitations were found in studies on the spatiotemporal evolution and influencing factors of economic development performance under carbon emission constraints. First, existing empirical studies primarily used the traditional DEA or super-efficiency DEA model [22]. However, the economic development performance scores obtained in these studies reflected relative performance, resulting in a lack of understanding of the overall change of dynamic performance. Although most researchers developed a comprehensive index system based on input and output, there is a prominent flaw in the process of index construction, which often overlooks the non-expected output index. Some researchers treat industrial sulfur dioxide, smoke dust, and solid waste as the non-expected output index, although the meaning of the non-expected output index is not comprehensive enough. Therefore, the research results of previous studies are not precise. Although related domestic studies are relatively mature, they focus on performance characterization and do not describe its inherent driving mechanism, resulting in the lack of pertinence and applicability of research results.

Therefore, this study observed 30 provinces in mainland China in 2008, 2012, 2016, and 2020 to evaluate China’s economic development performance under carbon emission constraints. It used the DEA model and Malmquist productivity index to conduct the evaluation. Furthermore, the spatiotemporal evolution characteristics of static and dynamic performance were analyzed. Finally, the influencing factors of economic development performance were discussed using the Tobit model, and the policy implications for the improvement of economic development performance were put forward.

## Methods

### Theoretical framework

**Fig. 1:**
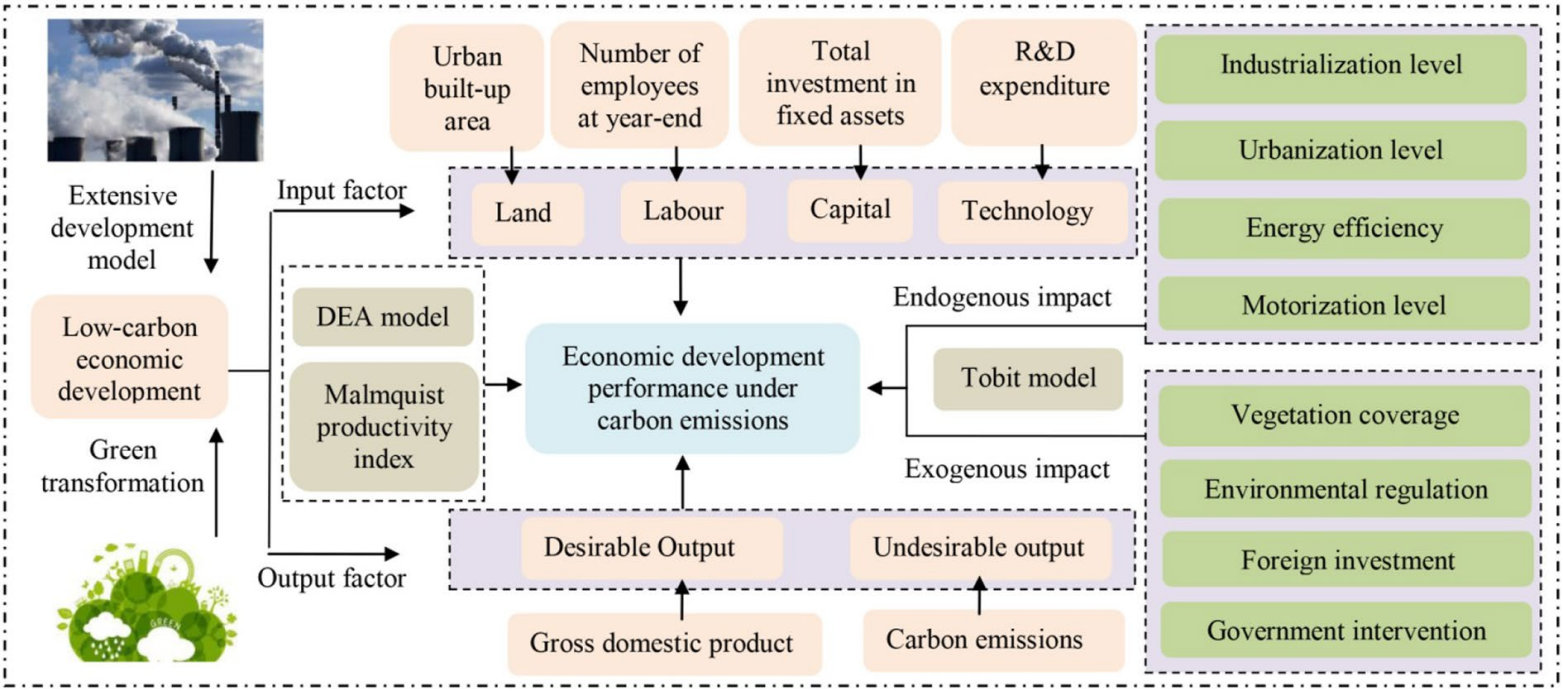
Theoretical framework

The traditional high-carbon economic development mode aims to maximize economic output and minimize the input of factors. Although this mode greatly accelerates industrialization and urbanization, it causes major environmental issues. The key to solving this problem lies in shifting from a high-carbon to a low-carbon economic development mode. Compared with the high-carbon economic development mode, the low-carbon mode aims to maximize economic output and reduce carbon emissions, while minimizing the input of factors [32]. At present, it is a huge challenge to reduce carbon emissions and promote economic development simultaneously. Based on the environmental externality theory, the sustainable development theory, and the connotation of the low-carbon economy, this study first constructed an index system to evaluate economic development performance under carbon emission constraints. This index system considers multiple input and output factors. Second, the DEA model and Malmquist productivity index were used to calculate technical efficiency, pure technical efficiency, scale efficiency, total factor productivity, technological change, scale efficiency change index, and pure technical efficiency change. Then, the study analyzed the spatiotemporal evolution characteristics of the static and dynamic performance of economic development. Finally, the evaluation index system of the factors influencing economic development performance was constructed in accordance with endogenous and exogenous variables. The Tobit model was used to discover the key influencing factors. In addition, strategies for optimizing and promoting the low-carbon economy in China were proposed (Fig. 1).

### Estimation of count data models

DEA is a method used to evaluate the efficiency of decision units that have multiple inputs and outputs. Assuming that there are *L* types of input and *M* types of output indicators, *x*_*jl*_ represents the input amount of the *L* types of resources, whereas *y*_*jm*_ represents the output amount of the *M* types of resources in *j* province. For the *n* (*n* = *1, 2…, K*) province, the formula is [33] as follows:

1 $$ \left\{ {\begin{array}{*{20}l}    {{\text{min}}\left( {\theta  - \varepsilon \left( {e_{1}^{T} s^{ - }  + e_{2}^{T} s^{ + } } \right)} \right);} \hfill  \\    {s.t.\sum\limits_{{j = 1}}^{K} {x_{{jl}} } \lambda _{j}  + s^{ - }  = \theta x_{l}^{n} ;\quad l = 1,2, \ldots L} \hfill  \\    {\sum\limits_{{j = 1}}^{K} {y_{{jm}} } \lambda _{j}  - s^{ + }  = y_{m}^{n} ;\quad m = 1,2, \ldots M;\quad n = 1,2, \ldots K} \hfill  \\   \end{array} } \right. $$

where *θ*(0 < *θ* ≤ 1) is the technical efficiency index; *λ*_*j*_ (*λ*_*j*_ ≥ 0) is the weight variable; *s*^*−*^(*s*^*−*^ ≥ 0)is the relaxation variable; *s*^+^(*s*^+^ ≥ 0) is the residual variable; *ε* is the non-Archimedean infinitesimal; and *e*_*1*_^*T*^ = (1, 1,…,1) ∈ E_m_ and *e*_*2*_^*T*^ = (1, 1,…,1) ∈ E_k_ are *m* and *k*-dimensional unit vector spaces, respectively. The closer *θ* is to 1, the higher the score for the *n* province. Equation 1 can be converted to a DEA model with variable returns to scale (*VRS*) by introducing the constraint condition ${\sum }_{j=1}^{k}{\lambda }_{j}=1$. Using the *VRS* model, technical efficiency can be decomposed into pure technical efficiency (*Vrste*) and scale efficiency (*Scale*).

Economic development performance under carbon emission constraints consists of static and dynamic performance. In particular, static performance explores the relative efficiency value of each evaluation unit within a given period, while dynamic performance reflects the sequential changes in the relative efficiency value of each evaluation unit. The efficiency value obtained by the DEA model can be used to measure the relative performance of each evaluation unit. When studying the change in the efficiency value in different periods, it is necessary to introduce the Malmquist productivity index. If (*x*_*t*_, *y*_*t*_) and (*x*_*t*+*1*_, *y*_*t*+*1*_) represent the input–output relationship in *t* and *t* + *1* periods, the change of the input–output relationship from (*x*_*t*_, *y*_*t*_) to (*x*_*t*+*1*_, *y*_*t*+*1*_) is the change of total factor productivity. *D*_*c*_^*t*^ (*x*^*t*^, *y*^t^) and *D*_*c*_^*t*+*1*^(*x*^*t*+*1*^, *y*^*t*+*1*^) are distance functions (Eqs. 3 and 4). The Malmquist productivity index based on *t* and *t* + *1* are [32]:

2 $$ M_{t} \left( {x^{t} ,y^{t} ,x^{t + 1} ,y^{t + 1} } \right) = \frac{{D_{c}^{t} \left( {x^{t + 1} ,y^{t + 1} } \right)}}{{D_{c}^{t} \left( {x^{t} ,y^{t} } \right)}} $$

3 $$ M_{t + 1} \left( {x^{t} ,y^{t} ,x^{t + 1} ,y^{t + 1} } \right) = \frac{{D_{c}^{t + 1} \left( {x^{t + 1} ,y^{t + 1} } \right)}}{{D_{c}^{t + 1} \left( {x^{t} ,y^{t} } \right)}} $$

According to the rational index, the geometric average value between the two is defined as the total factor productivity index. Its formula is as follows:

4 $$ M\left( {x^{t} ,y^{t} ,x^{t + 1} ,y^{t + 1} } \right) = \left( {M_{t} *M_{t + 1} } \right)^{1/2} = \left[ {\frac{{D_{c}^{t} \left( {x^{t + 1} ,y^{t + 1} } \right)*D_{c}^{t + 1} \left( {x^{t + 1} ,y^{t + 1} } \right)}}{{D_{c}^{t} \left( {x^{t} ,y^{t} } \right)*D_{c}^{t + 1} \left( {x^{t} ,y^{t} } \right)}}} \right]^{1/2} $$

Malmquist’s productivity can be decomposed into technical efficiency (*Effch*) and technological change (*Techch*) under the *VRS* hypothesis. Moreover, the change in technical efficiency can be decomposed into pure technical efficiency (*Pech*) and scale efficiency (*Sech*). Therefore, Eq. 4 becomes:

5 $$ \begin{aligned} M\left( {x^{t} ,y^{t} ,x^{t + 1} ,y^{t + 1} } \right) = \,& \frac{{D_{v}^{t} \left( {x^{t + 1} ,y^{t + 1} } \right)}}{{D_{v}^{t} \left( {x^{t} ,y^{t} } \right)}}*\left[ {\frac{{D_{v}^{t + 1} \left( {x^{t} ,y^{t} } \right)*D_{v}^{t} \left( {x^{t + 1} ,y^{t + 1} } \right)}}{{D_{v}^{t + 1} \left( {x^{t} ,y^{t} } \right)*D_{v}^{t + 1} \left( {x^{t + 1} ,y^{t + 1} } \right)}}} \right]^{\frac{1}{2}} \\ \quad \quad \quad \quad \quad \quad \quad \quad \quad & *\left[ {\frac{{\frac{{D_{c}^{t} \left( {x^{t + 1} ,y^{t + 1} } \right)}}{{D_{v}^{t} \left( {x^{t + 1} ,y^{t + 1} } \right)}}}}{{\frac{{D_{c}^{t} \left( {x^{t} ,y^{t} } \right)}}{{D_{v}^{t} \left( {x^{t} ,y^{t} } \right)}}}}*\frac{{\frac{{D_{c}^{t + 1} \left( {x^{t + 1} ,y^{t + 1} } \right)}}{{D_{v}^{t + 1} \left( {x^{t + 1} ,y^{t + 1} } \right)}}}}{{\frac{{D_{c}^{t + 1} \left( {x^{t} ,y^{t} } \right)}}{{D_{v}^{t + 1} \left( {x^{t} ,y^{t} } \right)}}}}} \right] = Techch{*}Pech{*}Sech \\ \end{aligned} $$

where *M*(*x*^*t*^*, y*^*t*^*, x*^*t*+*1*^*, y*^*t*+*1*^) is the total factor productivity; *Techch*, *Pech*, and *Sech* are the change indicators of technological change, pure technical efficiency, and scale efficiency, respectively (Eq. 5). When the change indicators of the total factor productivity, technological change, pure technical efficiency, and scale efficiency are greater than 1, it indicates a positive trend and vice versa.

Due to the truncation of the technical efficiency score value obtained by the DEA model, this study used the Tobit regression model to analyze the influencing factors of economic development performance under carbon emission constraints. Its formula [34] is as follows:

6 $$ Y_{i}^{*}  = \sum\nolimits_{{i\, = \,1}}^{n} {a_{i} } X_{i}  + b_{i} ;\;i = 1,2,3 \ldots n;\quad Y_{i}  = \left\{ {\begin{array}{*{20}l}    {Y_{i}^{*} ,}  \\    {0,}  \\   \end{array} } \right.\begin{array}{*{20}l}    {Y_{i}^{*}  > 0;}  \\    {Y_{i}^{*}  \le 0;}  \\   \end{array}   $$

where ${Y}_{i}^{*}$ is the dependent variable; *Y*_*i*_ is the technical efficiency index; *X*_*i*_ is the independent variable; *a*_*i*_ represents the coefficient; and *b*_*i*_ is the error term (Eq. 6).

### Data sources

#### Selection of evaluation indicators of economic development performance

In reference to relevant studies, this study selected input indicators based on land, labor, capital, and technology, and the output indicators are based on the economy and carbon emissions to construct the evaluation index system of economic development performance under carbon emission constraints [35, 36]. In particular, the urban built-up area, number of social employees at the end of the year, fixed asset investment, and research and development (R&D) expenditure represent input indicators, while gross domestic product (GDP) and carbon emissions represent output indicators (Table 1). The total carbon emissions index is an undesirable output index; therefore, it was subject to inverse transformation.

**Table 1:** Index system of economic development performance under carbon emission constraints

Index attribute	Index selection	Definition of Indicator
Input indicators	Urban built-up area/km^2^	Land input
	Number of employees at year-end/ten thousand	Labor input
	Total investment in fixed assets/100 million yuan	Capital input
	R&D expenditure/100 million yuan	Technology input
	Total carbon emission/t	Carbon emission pressure
Output indicators	Gross domestic product/100 million yuan	Economic output

#### Selection of factors influencing economic development performance

The progress and efficiency of the industrial structure directly determine the speed of economic development and carbon emissions [37]. Thus, this study chose the proportion of the output value of the secondary industry as the index representing the industrialization level. Urbanization indirectly affects carbon emissions through its impact on the distribution of economic and human activities [38]. This is why the urbanization rate was chosen as the measurement index. Moreover, energy efficiency and the number of motor vehicles can reflect the level of economic development and have a great impact on carbon emissions [5, 39]. Thus, this study chose energy consumption per unit of GDP to represent energy efficiency, and the number of civil motor vehicles owned by 10,000 people to represent the motorization level. Vegetation coverage, environmental regulation, and government intervention can reduce carbon emissions, while foreign investment has a strong impact on economic growth [32, 36]. This study chose the forest coverage rate, per capita SO_2_ emissions, actual foreign investment, and government general budget revenue to represent the aforementioned variables, respectively. Table 2 presents the index system of the influencing factors.

**Table 2:** Evaluation index system of influencing factors of economic development performance

Variable (abbreviated)	Meaning of variables	Specific indicators
Industrialization level (*IN*)	Reflect the overall level of industrial development	Proportion of output value of secondary industry (%)
Urbanization level (*UR*)	Reflect the overall level of urban development	Proportion of urban population in total population (%)
Energy efficiency (*EN*)	Reflect the efficiency of energy use	Energy consumption per unit of GDP (ton of standard coal/ten thousand yuan)
Vegetation coverage (*VE*)	Reflect the vegetation coverage	Forest coverage rate (%)
Environmental regulation (*ENV*)	Reflect comprehensive environmental protection efforts	Per capita SO_2_ emissions (t)
Motorization level (*MO*)	Reflect the circulation of economic activities	Number of civil motor vehicles owned by ten thousand people
Foreign investment (*FO*)	Reflecting the degree of openness	Actual foreign investment ($100 million)
Government intervention (*GO*)	Reflects the government’s ability to intervene in the economy	Government general budget revenue (100 million yuan)

#### Description of the data source

This study observed 30 provinces in mainland China (except Hong Kong, Macao, Taiwan, and Tibet) to evaluate China’s economic development performance under carbon emission constraints and analyze its influencing factors. The data were obtained from the China Statistical Yearbook of 2009, 2013, 2017, and 2021; China Energy Statistical Yearbook; and provincial statistical yearbooks. The data on carbon emissions were calculated using the methods provided by the Intergovernmental Panel on Climate Change (IPCC). The total amount of various types of energy consumption was multiplied by their average low calorific value and CO_2_ emission coefficient [40]. As direct data on energy consumption per unit of GDP of each province in 2016 and 2020 were unavailable, the study divided the total energy consumption of each province by its total GDP as a conversion method [41, 42].

## Results

### Evaluation of the static performance of economic development

#### Analysis of the static performance level

The software DEAP was used to measure the static performance of the economic development of 30 provinces based on the collected input–output index data. The technical efficiency of each province and its exponential decomposition results are presented in Table 3.

**Table 3:** Static economic development performance score under carbon emission constraints

Province	2008			2012			2016			2020			Average
	crste	vrste	scale	crste	vrste	scale	crste	vrste	scale	crste	vrste	scale	crste
Beijing	1.00	1.00	1.00	1.00	1.00	1.00	1.00	1.00	1.00	1.00	1.00	1.00	1.00
Tianjin	0.76	0.82	0.93	0.78	0.78	1.00	0.93	1.00	0.93	1.00	1.00	1.00	0.87
Hebei	0.50	0.67	0.74	0.48	0.52	0.93	0.52	0.52	1.00	0.48	0.53	0.91	0.50
Shanxi	0.55	0.56	0.99	0.57	0.63	0.91	0.67	0.70	0.95	0.78	0.86	0.91	0.64
Inner Mongolia	0.78	0.84	0.93	0.75	0.87	0.86	0.70	0.72	0.97	0.74	0.76	0.98	0.74
Liaoning	0.84	1.00	0.84	0.87	1.00	0.87	1.00	1.00	1.00	1.00	1.00	1.00	0.93
Jilin	1.00	1.00	1.00	1.00	1.00	1.00	1.00	1.00	1.00	1.00	1.00	1.00	1.00
Heilongjiang	1.00	1.00	1.00	1.00	1.00	1.00	1.00	1.00	1.00	1.00	1.00	1.00	1.00
Shanghai	0.63	0.66	0.95	0.88	0.89	0.99	0.61	0.64	0.95	0.70	0.71	0.98	0.71
Jiangsu	0.70	0.78	0.90	0.79	0.88	0.90	0.80	0.90	0.89	0.78	0.97	0.80	0.77
Zhejiang	0.68	0.72	0.95	0.76	0.76	1.00	0.74	0.76	0.98	0.90	0.77	0.90	0.77
Anhui	0.94	0.94	1.00	0.97	0.98	0.99	0.91	0.92	0.99	0.86	0.86	1.00	0.92
Fujian	0.59	0.59	1.00	0.67	0.68	0.98	0.64	0.65	0.99	0.61	0.62	0.99	0.63
Jiangxi	0.84	0.84	1.00	0.92	0.99	0.93	0.90	0.90	1.00	0.89	0.91	0.98	0.89
Shandong	0.65	0.88	0.74	0.70	0.85	0.82	0.75	0.90	0.83	0.80	1.00	0.80	0.73
Henan	0.60	1.00	0.60	0.66	0.95	0.69	0.64	0.74	0.87	0.70	0.80	0.88	0.65
Hubei	0.78	0.79	0.99	0.74	0.74	1.00	0.77	0.79	0.98	1.00	1.00	1.00	0.82
Hunan	0.61	0.61	1.00	0.60	0.60	1.00	0.59	0.59	1.00	0.64	0.64	1.00	0.61
Guangdong	1.00	1.00	1.00	1.00	1.00	1.00	1.00	1.00	1.00	1.00	1.00	1.00	1.00
Guangxi	0.77	0.89	0.87	0.80	0.84	0.95	0.81	0.86	0.94	0.80	0.81	0.99	0.80
Hainan	1.00	1.00	1.00	1.00	1.00	1.00	1.00	1.00	1.00	1.00	1.00	1.00	1.00
Chongqing	0.69	0.70	0.99	0.79	0.80	0.99	0.85	0.85	1.00	0.92	0.93	0.99	0.81
Sichuan	0.61	0.66	0.93	0.67	1.00	0.67	0.79	0.96	0.82	0.87	1.00	0.87	0.74
Guizhou	0.58	0.59	0.98	0.62	0.64	0.97	0.64	0.65	0.99	0.61	0.62	0.99	0.61
Yunnan	0.63	0.82	0.77	0.74	0.81	0.91	0.71	0.72	0.99	0.55	0.56	0.99	0.66
Shaanxi	0.55	0.55	1.00	0.56	0.56	1.00	0.58	0.59	0.98	0.57	0.58	0.99	0.57
Gansu	0.89	0.89	1.00	0.88	0.89	0.99	0.97	0.98	0.99	0.89	1.00	0.89	0.91
Qinghai	0.57	1.00	0.57	0.46	1.00	0.46	0.77	1.00	0.77	0.69	1.00	0.69	0.62
Ningxia	1.00	1.00	1.00	1.00	1.00	1.00	1.00	1.00	1.00	1.00	1.00	1.00	1.00
Xinjiang	1.00	1.00	1.00	1.00	1.00	1.00	1.00	1.00	1.00	1.00	1.00	1.00	1.00
Average	0.76	0.83	0.92	0.79	0.86	0.93	0.81	0.84	0.96	0.83	0.86	0.95	0.80

According to Table 3, China’s economic development performance under carbon emission constraints and its decomposition indices had the following characteristics:

First, the static performance level was generally low, and only a few provinces reached the optimal performance level of economic development. The technical efficiency index for 2008, 2012, 2016, and 2020 was 0.76, 0.79, 0.81, and 0.83, indicating that the static performance level of China’s economic development under carbon emissions constraint reached only 76%, 79%, 81%, and 83% of the optimal level, respectively. The average value of the technical efficiency index of China’s economic development in the given years was 0.80, indicating that the static performance reached 80% of the optimal level and there is great potential for improvement. In the future, the static performance level can be improved by optimizing the allocation of all input factors. In terms of provinces, the number of provinces with a technical efficiency index of 1 in 2008, 2012, 2016, and 2020 was 7, 7, 8, and 10, accounting for 23%, 23%, 27%, and 33% of the total number of research units, respectively. In particular, the technical efficiency index of Beijing, Jilin, Heilongjiang, Guangdong, Hainan, Ningxia, and Xinjiang was always 1, indicating that the static performance level reached the optimal level, and no input redundancy and output shortage were found in these provinces in those periods. However, the technical efficiency index of other provinces fluctuated and did not reach the optimal level in the given periods. This indicates that the static performance of most provinces can improve in the future.

Second, the pure technical efficiency index was higher than the technical efficiency index and slightly lower than the scale efficiency index. The pure technical efficiency index is the key to improving China’s economic development performance. In 2008, 2012, 2016, and 2020, the pure technical efficiency index was 0.83, 0.86, 0.84, and 0.86, respectively, indicating that pure technical efficiency reached 83%, 86%, 84%, and 86% of the optimal level. The average value of the pure technical efficiency index of China’s economic development in the four years was 0.85. This means that the pure technical efficiency reached 85% of the optimal level, indicating significant untapped potential. Specifically, the number of provinces with optimal pure technical efficiency in 2008, 2012, 2016, and 2020 was 10, 10, 10, and 14, accounting for 33%, 33%, 33%, and 47% of the total number of research units, respectively. The pure technical efficiency index of Beijing, Liaoning, Jilin, Heilongjiang, Guangdong, Hainan, Qinghai, Ningxia, and Xinjiang was always 1, meaning that the pure technical efficiency of these provinces was fully exploited during their economic development. In contrast, the pure technical efficiency index of other provinces fluctuated and failed to reach the optimal level. This indicates that the pure technical efficiency of these provinces was not fully exploited like with the ones mentioned above. The pure technical efficiency index of China’s provinces was slightly lower than the scale efficiency index. Based on this observation, it can be deduced that pure technical efficiency is the key to limiting the improvement of the static performance of China’s economic development. This conclusion is drawn based on the characteristics of technical efficiency and its decomposition index.

Finally, the number of provinces with the highest scale efficiency index was significantly larger than that with the highest technical efficiency index. Thus, leveraging scale efficiency is still an effective way to improve the performance of China’s economic development. In 2008, 2012, 2016, and 2020, the scale efficiency index was 0.92, 0.93, 0.96, and 0.95, indicating that scale efficiency reached 92%, 93%, 96%, and 95% of the optimal level, respectively. The average value of the scale efficiency in the given periods was 0.94, indicating that the potential of scale efficiency was fully exploited, reaching 94% of the optimal level. Particularly, the number of provinces with optimal scale efficiency in 2008, 2012, 2016, and 2020 was 13, 12, 12, and 12, which is higher than the technical efficiency index and pure technical efficiency index in the given years. The scale efficiency index of Beijing, Heilongjiang, Jilin, Hunan, Guangdong, Hainan, Ningxia, and Xinjiang was 1 in different years, indicating that there was no factor input redundancy and output insufficiency. However, the scale efficiency index of other provinces fluctuated in different years. It is suggested that these provinces should adjust the allocation ratio of various factors in time to avoid excessive or insufficient resource input. Remarkably, the scale efficiency index was relatively close to the optimal level, so the static performance can be improved to a certain extent by expanding the production scale.

#### Spatial differentiation characteristics of static performance

**Fig. 2:**
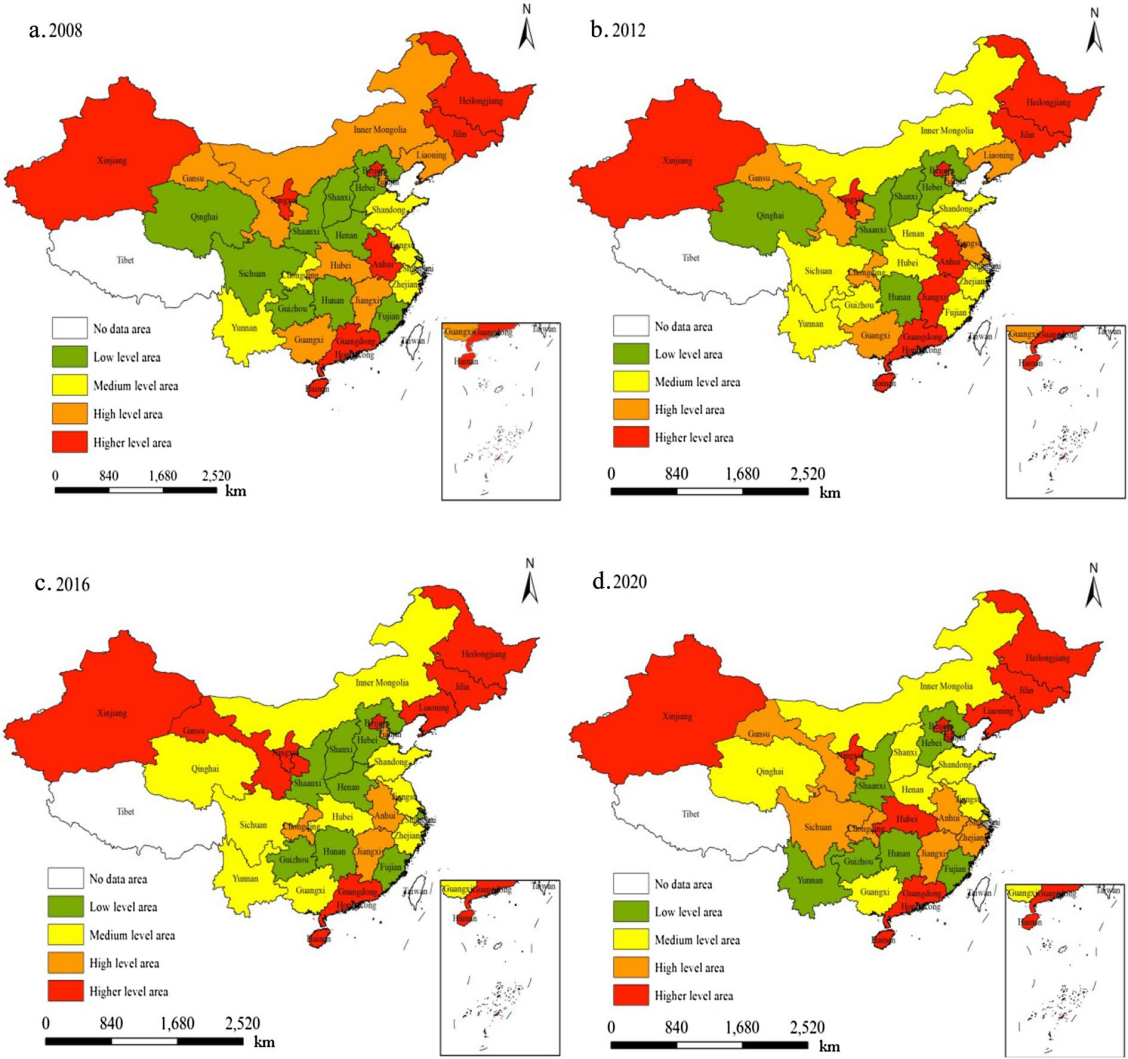
Spatial distribution of static performance of China’s economic development

The results of the static performance in 2008, 2012, 2016, and 2020 were classified into higher-, high-, medium- and low-level areas using the Jerks classification method, and their spatial distribution patterns were analyzed (Fig. 2).

Figure 2 depicts the spatial distribution characteristics of static performance in 2008, 2012, 2016, and 2020.

First, the static performance of economic development exhibited a trend of rising and then falling. The maximum value of the static performance of China’s economic development in the given years was 1. The provinces with low static performance were Hebei, Shaanxi, Guizhou, Hunan, and Qinghai, with an average value of a technical efficiency index of 0.50, 0.57, 0.61, 0.61, and 0.62, respectively. This indicates that it was only 50%, 57%, 61%, 61%, and 62% of the optimal level, which is lower than the national average level of 80% for the same period. This reveals that the static performance of China’s economic development was particularly prominent. When it comes to the spatial distribution of static performance, there were 8, 9, 9, and 10 provinces in the higher-level area and 7, 6, 4, and 7 provinces were found in the high-level area in 2008, 2012, 2016, and 2020, respectively. In the medium-level areas, there were 6, 10, 9, and 7 provinces while in the low-level areas, there were 9, 5, 8, and 6 provinces in 2008, 2012, 2016, and 2020, respectively. According to the spatial variation of higher- and high-level areas, the static performance of China’s economic development experienced an upward trend from 2008 to 2012 and a downward trend from 2012 to 2020.

Second, the distribution of provinces in different performance areas gradually stabilized over time, revealing a distinct pattern of staggered characteristics. From 2008 to 2012, 10 provinces changed their static performance area, which are Jiangxi, Chongqing, Jiangsu, Sichuan, Guizhou, Henan, Hubei, Zhejiang, Fujian, and Inner Mongolia. However, from 2012 to 2016, the number was 11—Liaoning, Gansu, Anhui, Jiangxi, Qinghai, Guangxi, Jiangsu, Shanghai, Henan, Guizhou, and Fujian. The number of provinces with changes in static performance area from 2016 to 2020 was 9, which are Hubei, Tianjin, Gansu, Sichuan, Zhejiang, Shanghai, Shanxi, Henan, and Yunnan. Thus, the static performance areas of economic development in China have changed over time, mainly in Henan, Hubei, Yunnan, Guangxi, Jiangsu, Zhejiang, and Shanghai, while that in other provinces remained stable when comparing their evolution characteristics to the former provinces.

The static performance areas tended to overlap, exhibiting a scattered distribution. However, the provinces have different reasons for their disparities in static performance. Henan, Hubei, and Sichuan have extensive land use patterns, limited capital input, low energy use efficiency, less advanced technology, and a large population, which caused great input redundancy in land, capital, technology, workforce, and great output deficiency in economic development. In these provinces, input and output were generally on a medium level, exhibiting imbalances, which limited the improvement of their static performance. Moreover, Yunnan and Guangxi are located in underdeveloped regions of China. They produce less carbon emissions from energy consumption and less input of land, labor, capital, and technology. Therefore, these provinces belonged to low-level imbalances in terms of factor input and output, which is the reason for their low static performance. In contrast, Shanghai, Jiangsu, and Zhejiang are situated in developed coastal areas with a strong economic foundation, advanced technology, and a high degree of efficient and intensive land use. However, these provinces consume great amounts of fossil fuel to develop economically, resulting in high carbon emissions. Therefore, in these provinces, all factors belonged to high-level imbalances, indicating that high carbon emissions are the reason for the low static performance in Shanghai, Jiangsu, and Zhejiang.

### Evaluation of the dynamic performance of economic development

To thoroughly analyze the change in China’s dynamic economic development performance in 2008, 2012, 2016, and 2020, this study also used DEAP to process the input–output index data. It also used the Malmquist productivity index to calculate the change in China’s dynamic performance of economic development under carbon emission constraints. The total factor productivity and its decomposition index are presented in Table 4.

**Table 4:** Dynamic economic development performance under carbon emission constraints

Province	2008–2012				2012–2016				2016–2020			
	techch	pech	sech	tfpch	techch	pech	sech	tfpch	techch	pech	sech	tfpch
Beijing	1.43	1.00	0.50	0.72	0.75	1.00	1.55	1.16	1.06	1.00	1.08	1.14
Tianjin	1.11	0.87	0.97	0.94	1.20	1.15	0.96	1.32	1.15	1.00	1.07	1.23
Hebei	0.77	0.78	1.27	0.76	0.87	1.00	1.07	0.93	1.08	1.01	0.91	0.99
Shanxi	0.73	1.11	0.94	0.75	0.83	1.11	1.04	0.95	0.90	1.24	0.96	1.08
Inner Mongolia	0.97	1.00	0.93	0.90	0.99	0.81	1.15	0.92	1.11	1.05	1.01	1.18
Liaoning	1.04	1.00	0.99	1.03	1.32	1.00	1.09	1.43	0.89	1.00	1.00	0.89
Jilin	0.94	1.00	1.00	0.94	0.89	1.00	1.00	0.89	1.10	1.00	1.00	1.10
Heilongjiang	0.75	1.00	1.00	0.75	0.85	1.00	1.00	0.85	1.13	1.00	1.00	1.13
Shanghai	1.04	1.06	1.00	1.10	1.08	0.72	0.99	0.77	1.03	1.16	1.00	1.20
Jiangsu	1.07	1.11	0.99	1.17	0.92	1.02	1.02	0.96	1.10	1.08	0.89	1.05
Zhejiang	1.03	1.04	0.99	1.06	0.87	0.99	1.01	0.87	1.06	0.99	0.96	1.01
Anhui	1.08	1.02	1.01	1.11	0.81	0.93	1.00	0.75	1.02	0.93	1.01	0.96
Fujian	1.00	1.07	1.00	1.06	0.81	0.97	1.03	0.80	1.06	1.02	1.00	1.08
Jiangxi	1.10	1.10	0.99	1.20	0.77	0.91	1.08	0.75	1.03	1.00	0.99	1.02
Shandong	0.91	0.97	1.09	0.95	0.88	1.06	1.01	0.93	1.08	1.11	0.99	1.19
Henan	0.88	0.95	1.15	0.96	0.81	0.77	1.25	0.79	1.03	1.09	1.02	1.14
Hubei	0.82	0.94	1.01	0.78	0.83	1.06	0.98	0.86	1.05	1.27	1.02	1.37
Hunan	0.82	0.97	1.02	0.81	0.80	0.97	1.00	0.78	1.02	1.10	0.99	1.11
Guangdong	0.95	1.00	1.00	0.95	0.83	1.00	1.00	0.83	0.97	1.00	1.00	0.97
Guangxi	0.84	0.95	1.08	0.87	0.80	1.02	0.99	0.81	0.99	0.94	1.06	0.99
Hainan	0.39	1.00	1.00	0.39	0.91	1.00	1.00	0.91	0.95	1.00	1.00	0.95
Chongqing	0.96	1.14	1.00	1.10	0.88	1.06	1.00	0.93	1.03	1.13	0.99	1.15
Sichuan	0.83	1.52	0.72	0.91	0.80	0.96	1.22	0.94	1.02	1.04	1.12	1.18
Guizhou	0.66	1.09	0.99	0.71	0.82	1.02	1.02	0.86	1.05	0.97	0.99	1.01
Yunnan	0.61	0.98	1.18	0.70	0.85	0.89	1.09	0.82	1.04	0.80	0.99	0.83
Shaanxi	0.96	1.01	1.00	0.97	0.88	1.05	0.98	0.91	1.08	1.00	1.00	1.08
Gansu	0.70	0.98	1.00	0.69	0.84	1.11	1.00	0.93	1.03	1.02	0.90	0.94
Qinghai	0.65	1.00	0.80	0.52	0.90	1.00	1.70	1.53	1.12	1.00	0.89	0.99
Ningxia	0.78	1.00	1.00	0.78	0.87	1.00	1.00	0.87	1.06	1.00	1.00	1.06
Xinjiang	0.66	1.00	1.00	0.66	0.88	1.00	1.00	0.88	1.09	1.00	1.00	1.09
Average	0.86	1.02	0.98	0.85	0.88	0.98	1.06	0.92	1.04	1.03	0.99	1.06

Table 4 indicates that the total factor productivity of China’s economic development performance under carbon emission constraints from 2008 to 2012 was only 0.85, indicating that the dynamic performance level declined by 15%. The technological change index, pure technical efficiency index, and scale efficiency index were 0.86, 1.02, and 0.98, respectively. This indicates that technological progress decreased by 14%, pure technical efficiency increased by 2%, and scale efficiency decreased by 2% from 2008 to 2012. During this period, the impact of technological progress on dynamic performance became limited, while the effect of scale efficiency became stronger. Therefore, technological degradation played a crucial role in the decline in the dynamic performance of economic development.

From 2012 to 2016, the total factor productivity index was 0.92, with an overall decrease of 8%. The technological change index, pure technical efficiency index, and scale efficiency index were 0.88, 0.98, and 1.06, respectively. Compared with the results in 2012, technological progress and pure technical efficiency in 2016 decreased by 12% and 2%. Based on the results, technological degradation was the main reason for the improvement of the dynamic economic development performance in this period.

Furthermore, the total factor productivity index from 2016 to 2020 was 1.06, indicating that the overall dynamic performance level of China’s economic development increased by 6%. The technological change index, pure technical efficiency index, and scale efficiency index were 1.04, 1.03, and 0.99, respectively. This indicates that China’s technological progress and pure technical efficiency increased by 4% and 3%, respectively, while scale efficiency decreased by 1%. Based on these results, technological progress and pure technical efficiency were the reasons for improved dynamic performance. It should be noted that scale efficiency drove the improvement of dynamic performance at one point, but it then became a constraint in a later period.

From 2008 to 2012, there were 8 provinces with a total factor productivity index greater than 1, namely Liaoning, Shanghai, Jiangsu, Zhejiang, Anhui, Fujian, Jiangxi, and Chongqing. This indicates that the dynamic performance of economic development in the mentioned provinces increased from 3 to 20%, exhibiting an upward trend. In contrast, the total factor productivity index of other provinces was less than 1, indicating that the dynamic performance of these provinces exhibited a downward trend, with a decrease from 3 to 61%. In addition, there were 21 provinces with a technological change index of less than 1, 9 with a pure technical efficiency index of less than 1, and 11 with a scale efficiency index of less than 1. This means that technological progress was the primary factor affecting the improvement of dynamic performance, followed by scale efficiency. Obviously, pure technical efficiency had little influence on the dynamic performance of economic development in this period.

From 2012 to 2016, there were only 4 provinces with a total factor productivity index greater than 1, namely Beijing, Tianjin, Liaoning, and Qinghai. This indicates that the dynamic performance in these provinces had an upward trend. In 2016, the total factor productivity index in other provinces was less than 1, indicating that the dynamic performance of most provinces had a downward trend compared with the results in 2012. During this period, the number of provinces with a technological change index, pure technological efficiency index, and scale efficiency index less than 1 was 27, 10, and 5, respectively, indicating that technological progress and scale efficiency had a greater impact on the dynamic performance of economic development, while pure technological efficiency had a smaller impact.

From 2016 to 2020, the number of provinces with a total factor productivity index greater than 1 was 21, namely Beijing, Tianjin, Shanxi, Inner Mongolia, Jilin, Heilongjiang, Shanghai, Jiangsu, Zhejiang, Fujian, Jiangxi, Shandong, Henan, Hubei, Hunan, Chongqing, Sichuan, Guizhou, Shaanxi, Ningxia, and Xinjiang. This indicates that the dynamic performance of these provinces increased. Most of the provinces exhibited rapid growth, with an increase of more than 8%. The number of provincial units with the technological change index, pure technical efficiency index, and scale efficiency index less than 1 was 5, 5, and 11, respectively, indicating that scale efficiency hinders the improvement of dynamic performance.

### Analysis of the influencing factors of economic development performance

To mitigate the interference caused by data heteroscedasticity in the regression model, the original independent variables data underwent a logarithmic transformation. This study used the Tobit model to analyze the influencing factors of the static performance of China’s economic development in 2008, 2012, 2016, and 2020. The results are presented in Table 5.

**Table 5:** Analysis of influencing factors of economic development performance using the Tobit model

Variable	2008		2012		2016		2020	
	Coefficient	Prob	Coefficient	Prob	Coefficient	Prob	Coefficient	Prob
Ln IN	− 0.0929	0.6131	− 0.3143	0.0903	− 0.1780	0.2008	− 0.2014	0.2470
Ln UR	0.6391	0.0023***	0.8971	0.0348**	1.0350	0.0113**	1.3827	0.0012***
Ln EN	0.0749	0.6362	− 0.3216	0.0757*	− 0.4744	0.0256**	− 0.3156	0.0501*
Ln VE	0.0081	0.8118	− 0.0080	0.8521	0.0129	0.0957*	0.0139	0.0685*
Ln ENV	− 0.1426	0.1228	0.1099	0.2937	0.0309	0.7340	0.0711	0.3381
Ln MO	− 0.1051	0.1456	− 0.1261	0.2135	0.1416	0.7569	0.1014	0.4312
Ln FO	− 0.0819	0.0920*	− 0.0798	0.3107	− 0.0531	0.4491	− 0.0345	0.3381
Ln GO	0.0277	0.6965	0.0197	0.8353	− 0.0571	0.4551	− 0.0615	0.6225
C	− 0.3825	0.8451	2.2332	0.2204	1.7607	0.1517	1.9672	0.1232
Log-likelihood	18.2935	–	17.9547	–	17.8675	–	16.8539	–
AIC	− 0.5528	–	− 0.5303	–	− 0.5912	–	− 0.4569	–
SC	− 0.0858	–	− 0.0632	–	− 0.1129	–	− 0.0101	–

^***^Denotes significance at the 1% level

^**^Denotes significance at the 5% level

^*^Denotes significance at the 10% level

– indicates that the item does not exist

As presented in Table 5, the absolute values of AIC and SC in the 2008, 2012, 2016, and 2020 models were all less than 1, while the log-likelihood value was high. Therefore, according to the values of model evaluation criteria, the Tobit model was effective. Specifically, the urbanization level and foreign investment in 2008 passed the significance test at the 1% and 10% levels, respectively, while other independent variables did not pass the significance test at the 10% level. This result indicates that the urbanization level and foreign investment were the main factors affecting China’s economic development performance under carbon emission constraints in 2008, while the influence of other factors was not clear. In 2012, urbanization level and energy efficiency passed the significance test at the 5% and 10% levels, respectively. The influence of other variables on economic development performance was not clear yet again, indicating that urbanization level and energy efficiency were the main factors affecting economic development performance in 2012. The number of variables in the Tobit model increased from 2 in 2012 to 3 in 2016, including vegetation coverage, urbanization level and energy efficiency. These variables were significant at the 10%, 5%, and 5% levels, respectively. This indicates that urbanization level, energy efficiency, and vegetation coverage were the main factors affecting economic development performance in 2016, while the influence of factors such as motorization level, environmental regulation, and government intervention was not obvious. In 2020, the number of variables in the model remained the same. These variables were quite consistent with the direction of economic development performance. There were slight variations in the significance level and intensity of the effect of the variables on economic development performance. In particular, urbanization level was significant at the 1% level, while energy efficiency and vegetation coverage were significant at the 10% level. The impact of urbanization level and vegetation coverage on economic development performance gradually increased, while the impact of energy efficiency decreased when compared with results in 2016.

The results indicate that urbanization level was the main factor affecting economic development performance under carbon emission constraints. The influence of this factor increased from 0.6391 in 2008 to 1.3827 in 2020, with an average annual increase of 0.0619 units. It was found that urbanization promoted the rapid agglomeration of population, industry, capital, technology, and other factors in urban areas, which created favorable conditions for attracting substantial fixed asset investment and caused the influx of rural labor. This result can greatly contribute to the improvement of land use and energy use efficiency; the transformation of production and consumption; and the introduction and promotion of advanced production, energy saving, and environmental protection technology. Similarly, the result can lead to rapid economic development and a significant reduction in carbon emissions, thereby achieving a low-carbon economy and green development at the national level.

However, energy efficiency, vegetation coverage, and foreign investment were also important factors affecting economic development performance, among which energy efficiency had the greatest impact. Low energy efficiency causes people to consume more energy in the production process. The current energy structure in China is powered by fossil fuel. Due to this structure, low energy efficiency leads to carbon emissions, hindering the improvement of economic development performance. Vegetation coverage also affects economic development performance. Through photosynthesis, plants, especially trees, have the ability to convert carbon dioxide into biomass and release oxygen, thus reducing carbon emissions and alleviating the environmental load caused by human activities. Therefore, increasing vegetation coverage can improve economic development performance. The impact of foreign investment on economic development performance is evident in the fact that the more open the region, the more foreign capital, talents, and advanced technology it receives. The introduction of capital, talent, and technology can promote the optimization and upgrade of the regional energy and industrial structure and the rapid development of the regional economy. Thus, carbon emissions can be effectively reduced, and economic development performance can be improved.

## Discussions

### Comparison with similar studies

The results of the economic development performance in this study were similar to that of other related studies. The average scores of China’s technical efficiency in 2005, 2010, 2015, and 2019 were 0.87, 0.89, 0.80, and 0.85 [36], respectively, which are slightly higher than the static economic development performance scores in this study. However, the spatial distribution characteristics of technical efficiency in China in other studies were very similar to those in this one. The energy structure and foreign investment are the main factors affecting the static performance of economic development. Similar research results indicate that improving energy efficiency; reducing the use of fossil fuels; and introducing foreign capital, technology, and talents are important ways to promote China’s economic development and reduce carbon emissions. The results of the DEA model in other studies indicate that the overall level of low-carbon economy in China was not high and its performance in most provinces was not optimal. The pure technical efficiency index was lower than the scale efficiency value. Therefore, pure technical efficiency is the main factor hindering the development of a low-carbon economy [43]. This conclusion is consistent with the results of this study, which indicates that advanced technology plays a greater role in economic development performance than expanding the production scale. The dynamic performance of China’s low-carbon economy increased significantly from 2000 to 2010 but then decreased from 2010 to 2014. During these ten years, the dynamic performance of China’s low-carbon economy shifted from pure technical efficiency and scale efficiency to technical progress [32]. In this study, the dynamic performance of economic development also has similar characteristics, which corroborates the idea that China’s low-carbon economy is improving. In addition, both the above studies and this study have confirmed that vegetation coverage is an important factor affecting economic development performance. This means that increasing afforestation and green space per capita is an important way to improve economic development performance and carbon absorption capacity. The difference between the studies is that the factors hindering dynamic performance gradually shift from technological progress to scale efficiency, indicating that scale management can significantly improve economic development performance under carbon emission constraints. Other studies have confirmed that green innovation affects carbon emission through the energy consumption structure effect, industrial structure effect, urbanization effect, and foreign direct investment effect [5, 44]. This is consistent with the results of the influencing factors in this study, indicating that developing clean energy, promoting industrial structure upgrade and people-oriented urbanization level, and attracting more foreign investment are effective means to improve economic development performance.

### Limitations of this study

This study analyzed the spatiotemporal evolution characteristics and influencing factors of economic development performance under carbon emission constraints. However, it has its limitations.

First, the study adopted the traditional DEA model and Malmquist productivity index to investigate the spatiotemporal variation characteristics of economic development performance under carbon emission constraints. However, a limitation of the traditional DEA model is its inability to further decompose when the technical efficiency scores of multiple evaluation units reach the optimal level simultaneously. Therefore, the final result is relatively imprecise. Although the Malmquist productivity index can accurately reflect the changing characteristics of dynamic performance, it is based on the traditional DEA model. There is an urgent need to modify the DEA model and combine the modified version with the Malmquist productivity index to more accurately depict the static and dynamic performance of economic development.

Second, the construction of the input–output index system was a complex and systematic project because it involved both quantitative and qualitative indices, such as regional development strategy, government management ability, business environment, and enterprise competitiveness. This study primarily relied on the data availability principle to construct the evaluation index system of economic development performance and influencing factors. However, it overlooked the significance of difficult quantitative indicators or factors, resulting in biased results of the evaluation. This undermines the applicability of the research results. Therefore, future research should construct a more scientifically-based and comprehensive index system to reflect high-quality economic development.

Finally, the study chose only 8 factors as endogenous and exogenous variables to investigate the direction and intensity of the influencing factors on economic development performance under carbon emission constraints. Factors other than the ones given in this study and the possible effect of their spatial scale were not considered, resulting in the lack of pertinence and applicability of the final research results. In addition, the Tobit model adopted in the study belongs to the group of traditional econometric models. This means that it overlooks the spatial dependence between variables, which questions the accuracy of the regression results. Therefore, it is necessary to use a spatial econometric model to conduct a multi-scale analysis of influencing factors of economic development performance under carbon emission constraints in the future.

## Conclusions

This study used the DEA model, Malmquist productivity index, and Tobit model to analyze the spatiotemporal evolution characteristics and influencing factors of China’s economic development performance under carbon emission constraints. The main findings are as follows:

1. Overall, the static performance of China’s economic development increased. The differences in the static performance were prominent, exhibiting a development pattern of staggered characteristics. The technical efficiency index of China’s economic development in 2008, 2012, 2016, and 2020 was 0.76, 0.79, 0.81, and 0.83, respectively. Therefore, the static performance reached only 76%, 79%, 81%, and 83% of the optimal level. This means that static performance has great potential for improvement. In terms of spatial distribution, static performance was dominant in the higher and high-level areas, which represents centralized and contiguous development.
2. The dynamic performance of economic development first declined and then rose. In the given periods, the dynamic performance of most provinces shifted from being constrained by technological progress to being constrained by scale efficiency. Moreover, the dynamic performance decreased by 15% from 2008 to 2012 and 8% from 2012 to 2016 but increased by 6% from 2016 to 2020, corroborating the trend of declining and then rising. In addition, the number of provinces with a technological changes index, pure technical efficiency index, and scale efficiency index less than 1 changed from 21, 9, and 11 in 2008 to 5, 5, and 11 in 2020, respectively, indicating that the scale efficiency index hindered dynamic performance.
3. Urbanization level, energy efficiency, vegetation coverage, and foreign investment were the main factors affecting economic development performance, while industrialization level, environmental regulation, and government intervention had no significant impact. Specifically, urbanization level always had the most positive effect on economic development performance, followed by energy efficiency, while vegetation coverage and foreign investment had a relatively small impact on economic development performance during the study period.

## Data Availability

The dataset supporting the conclusions of this article is included within the article.
